# Fundamental Frequency Variation of Neonatal Spontaneous Crying Predicts Language Acquisition in Preterm and Term Infants

**DOI:** 10.3389/fpsyg.2017.02195

**Published:** 2017-12-22

**Authors:** Yuta Shinya, Masahiko Kawai, Fusako Niwa, Masahiro Imafuku, Masako Myowa

**Affiliations:** ^1^Graduate School of Education, Kyoto University, Kyoto, Japan; ^2^Japan Society for the Promotion of Science, Tokyo, Japan; ^3^Department of Pediatrics, Graduate School of Medicine, Kyoto University, Kyoto, Japan

**Keywords:** crying, melody, preterm birth, low-birth-weight, language, fundamental frequency, vocalization

## Abstract

Spontaneous cries of infants exhibit rich melodic features (i.e., time variation of fundamental frequency [*F_0_*]) even during the neonatal period, and the development of these characteristics might provide an essential base for later expressive prosody in language. However, little is known about the melodic features of spontaneous cries in preterm infants, who have a higher risk of later language-related problems. Thus, the present study investigated how preterm birth influenced melodic features of spontaneous crying at term-equivalent age as well as how these melodic features related to language outcomes at 18 months of corrected age in preterm and term infants. At term, moderate-to-late preterm (MLP) infants showed spontaneous cries with significantly higher *F_0_* variation and melody complexity than term infants, while there were no significant differences between very preterm (VP) and term infants. Furthermore, larger *F_0_* variation within cry series at term was significantly related to better language and cognitive outcomes, particularly expressive language skills, at 18 months. On the other hand, no other melodic features at term predicted any developmental outcomes at 18 months. The present results suggest that the additional postnatal vocal experience of MLP preterm infants increased *F_0_* variation and the complexity of spontaneous cries at term. Additionally, the increases in *F_0_* variation may partly reflect the development of voluntary vocal control, which, in turn, contributes to expressive language in infancy.

## Introduction

Crying is one of the few means by which human infants can communicate a variety of emotions, including hunger, pain, and frustration. The majority of research investigating infant crying has evaluated acoustic features as a non-invasive index of neurophysiological states during the neonatal period, including pain stress (Stevens et al., 2007) and medical complications (Wasz-Höckert et al., 1985). In line with these findings, the most common acoustic feature of pain-induced cries is that of fundamental frequency (*F_0_*); for example, abnormally high *F_0_* values are related to a variety of medical conditions including chromosomal, endocrine, metabolic, and neurological disturbances (Wasz-Höckert et al., 1985).

Previous studies investigating preterm infants (gestational age < 37 weeks) have focused on *F_0_* in neonatal cries. Preterm infants exhibit higher *F_0_* of pain-induced cries before term-equivalent age compared to term newborns but these differences disappear around term-equivalent age (Michelsson et al., 1983; Johnston et al., 1993; Cacace et al., 1995). In contrast, the present authors found that the spontaneous cries (i.e., cries unaffected by external stress) of preterm infants at term-equivalent age are higher in terms of *F_0_* min, *F_0_* mean, and *F_0_* max (see section “Materials and Methods”) than those of term newborns (Shinya et al., 2014). Our research group also showed that shorter gestational age is significantly associated with higher *F_0_*, regardless of body size at cry recording and intrauterine growth retardation [IUGR]. Thus, rather than being a product of a smaller body size, the increased *F_0_* of spontaneous cries in preterm infants may be the result of more complicated neurophysiological states, such as low vagal activity (Shinya et al., 2016), due to their different intra- and extrauterine experiences.

**FIGURE 1:**
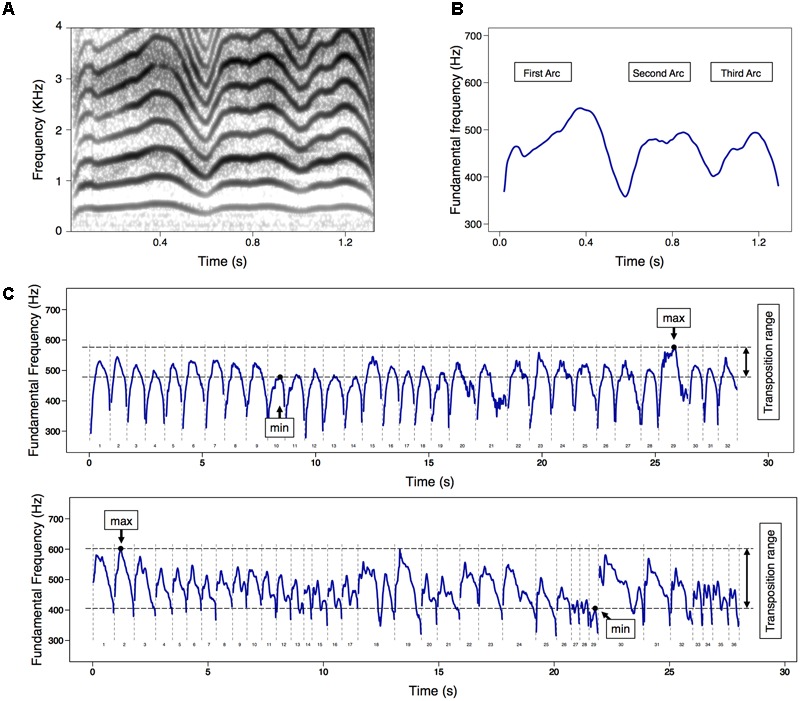
**(A)** An example of spectrogram and **(B)** corresponding melody (i.e., time series of fundamental frequency [*F_0_*]) contour diagrams for a complex cry with multiple-arcs. **(C)** Two examples of melody contours in a series of spontaneous cries in 60 s, indicating the *F_0_* transposition range, which means the difference between minimum and maximum values among *F_0_* max. The upper diagram indicated a smaller *F_0_* transposition range (98 Hz [478–576], 3.24 semitones), while the bottom one indicated a greater *F_0_* transposition range (197 Hz [405–602], 6.85 semitones). Not included in the diagrams were cry utterances excluded from the main analysis, respiratory phases, and no-phonatory periods.

On the other hand, melody (i.e., time variation in *F_0_*) is considered to be one of the most crucial acoustic features of infant cries. In term infants, spontaneous cries exhibit rich melodic features even during the neonatal period (Mampe et al., 2009; Wermke et al., 2016) and their cry melodies are reported to become increasingly more variable (Prescott, 1975) and complex (Wermke et al., 2002, 2007) across the first months of life. For example, developmental changes have been observed in the spontaneous cries of term infants such that *F_0_* variability (i.e., standard deviation [*SD*]) within a cry utterance increases from 28 Hz at 1–2 weeks of age to 53 Hz at 6–9 months of age (Prescott, 1975). Furthermore, the spontaneous cries of term infants show unidirectional development from mostly simple single-arc melody structures to an increasing number of multiple-arc structures during the first several months (Wermke et al., 2002, 2007; **Figures 1A,B**). These developmental changes in infant cry melody are assumed to reflect increases in laryngeal vocal coordination due to maturation in the developing central nervous system (CNS).

Broadening the perspective, there is a potential relationship between the development of an infant’s cry melody and language acquisition (Darwin, 1872; Lieberman, 1985; Wermke and Mende, 2009). The evolutionary viewpoint emphasizes that expressive language may originate from the imitation and modification of humans’ instinctive cries by articulate sounds (Darwin, 1871). According to this view, an increased melodic variation in infant crying is assumed to provide an essential base for linguistic prosody expressive of various emotions (Darwin, 1872; Jespersen, 1922; Wermke and Mende, 2009). This assumption is consistent with observations of the high sensitivity to melodic features of adult speech during the fetal (Kisilevsky et al., 2009; Partanen et al., 2013; Webb et al., 2015) and neonatal (Kujala et al., 2004; Naoi et al., 2013) periods. Additionally, infant cry melody is shaped by ambient language during these periods (Mampe et al., 2009; Wermke et al., 2016).

The continuity between cry melody and language development is supported by phonetic evidence as well. Wermke et al. (2007) reported that a high percentage of term infants who exhibit multiple-arc melody in cries made during the second month have better language outcomes at 2.5 years (Wermke et al., 2007). Previous studies of mammals suggest that the production of infant cries in humans involves cortical regions, such as the anterior cingulate gyrus, as well as brainstem regions (Müller-Preuss and Jürgens, 1976; Jürgens, 2002; Newman, 2007). Considering the rapid maturation of the CNS during the first few months of life, the development of infant cry melody may reflect increasing voluntary vocal control by the cortex, which provides experience that facilitates control over later non-cry vocalizations related to language (Lieberman, 1985; Wermke et al., 2007).

However, the manner in which preterm birth might affect the melodic features of spontaneous cries and how these cries are related to later language acquisition in preterm peers remain unclear. Preterm infants exhibit slower acquisition of receptive and expressive language skills in their second year of life (Foster-Cohen et al., 2007; Sansavini et al., 2011) as well as during childhood (Luu et al., 2009; Barre et al., 2011; van Noort-van der Spek et al., 2012). Furthermore, during the earlier stages of language acquisition, preterm infants exhibit atypical developmental patterns of preverbal communication, such as an earlier onset of canonical babbling (Eilers et al., 1993) and reductions in joint attention (Garner et al., 1991; De Schuymer et al., 2011a; Imafuku et al., 2017). Considering their atypical developmental pattern of language acquisition, the identification of melodic features of spontaneous cries during early developmental periods and the assessment of the relationships of these features with early language acquisition are significant issues (Smith and Ulvund, 2003; Peña et al., 2010; De Schuymer et al., 2011b).

Thus, the present study assessed melodic features of spontaneous cries in preterm infants with no severe complications at a term-equivalent age and compared these features to those of term newborns (Shinya et al., 2014). In particular, the variability (i.e., height of *F_0_* variation) and complexity (i.e., amount of melody arcs) of the melodic features were a specific focus. Furthermore, based on the assumption of a potential link between cry melody and language development (Darwin, 1872; Lieberman, 1985; Wermke and Mende, 2009), the manner in which the analyzed melodic features of spontaneous cries at term contribute to early communication and language development at 18 months of corrected age were examined in preterm and term infants. Especially, it was expected that infants who exhibit more variable and complex cry melodies at term would show better language outcomes at 18 months. We focused on their language outcomes at 18 months of age because it has been reported that preterm infants (gestational age ≤ 32 weeks) begin to show lower receptive and expressive language development by the age, compared to term infants (Sansavini et al., 2011).

## Materials and Methods

### Participants

The present study included 77 preterm infants (gestational age < 37 weeks) and 30 term infants (gestational age ≥ 37 weeks) who were recruited between 2011 and 2015 from Kyoto University Hospital. The preterm infants were assigned to two subgroups according to gestational age: very preterm (VP) infants (gestational age < 32 weeks; *n* = 36) and moderate-to-late preterm (MLP) infants (gestational age ≥ 32 weeks but < 37 weeks; *n* = 41); all participants were included in previous studies conducted by our research group (i.e., the preterm group in Shinya et al., 2014, 2016 and the term group in Shinya et al., 2016). The exclusion criteria consisted of severe neurological complications such as brain lesions (including periventricular leukomalacia, and Grade III or IV intraventricular hemorrhages), chromosomal abnormalities, and/or medical treatment required for respiratory disease at term-equivalent age. When all participants were in the hospital before cry recording, written informed consent was obtained from their parents. All participants came from Japanese families, and all families were considered middle class. On the basis of our interactions with the parents, all parents were regarded to be healthy with no cognitive and language deficits. The study was conducted with the approval of the ethics committee of Kyoto University Graduate School and Faculty of Medicine (No. E581) and was conducted according to standards specified in the Declaration of Helsinki from 1964. The demographic data of the participants at term-equivalent age are provided in **Table 1**.

**Table 1:** Demographic and cry melodic variables in preterm and term infants at term-equivalent age.

	Preterm infants						Term infants					
	VP (*n* = 36)			MLP (*n =* 41)			Term (*n =* 30)					
	*M*	*SD*	*Range*	*M*	*SD*	*Range*	*M*	*SD*	*Range*	*F*-Value	*p*-Value	*Post hoc* (*p* < 0.05)
**Demographic variables**
Gestational age (weeks)	28.5	2.3	23.1–31.9	34.6	1.4	32–36.9	39.5	1.1	37.1–41.4	353.93	<0.001	VP < MLP < Term
Birth weight (g)	1049	303	618–1640	1823	307	1308–2572	2903	358	2352–4110	273.42	<0.001	VP < MLP < Term
Apgar score 5 min	7.1	2.4	1–10	8.5	1.5	3–10	9.2	0.5	8–10	13.56	<0.001	VP < MLP, VP < Term
Postnatal age (days)	80.2	21.6	38–124	33.7	11.3	12–53	3.8	0.9	3–7	239.38	<0.001	VP > MLP > Term
Post-menstrual age (weeks)	39.9	1.3	37.3–42	39.4	1.0	37–41.9	40.1	1.0	38–41.9	3.88	0.0237	MLP < Term
Weight (g)	2744	386	1946–3602	2455	314	1946–3530	2770	370	2222–4028	9.11	<0.001	VP > MLP, MLP < Term
IUGR	10/36 (28%)			19/41 (41%)			2/30 (7%)					
Female	13/36 (36%)			29/41 (71%)			17/30 (57%)					
**Cry melodic variables**
*F_0_* range (Hz)	162	39	95–275	179	44	97–313	144	30	86–215	7.10	0.001	MLP > Term
*F_0_* sigma (Hz)	40	10	20–64	44	12	22–85	35	8	22–53	7.41	0.001	MLP > Term
*F_0_* transposition range (Hz)	177	50	101–265	199	69	76–375	159	42	83–235	4.55	0.013	MLP > Term
MCI ratio	0.29	0.16	0.04–0.63	0.34	0.17	0.03–0.8	0.29	0.13	0.09–0.55	1.03	0.362	
MCI number	1.12	0.38	0.38–1.84	1.30	0.34	0.43–2.38	1.10	0.30	0.64–1.78	4.16	0.018	MLP > Term

*VP, very preterm; MLP, moderate to late preterm; IUGR, intrauterine growth retardation; MCI, melody complexity index.*

### Procedure

#### Recording of Spontaneous Crying at Term-Equivalent Age

All infants were at term-equivalent age and studied between 5 and 9 p.m. while in a supine position in an open crib. Preterm infants were assessed in a growing care unit at Kyoto University Hospital where they stayed until leaving the hospital whereas term infants were assessed in a quiet examination room at the hospital. Environmental conditions, including the crib, noise level, and ambient temperatures, were controlled for all participants. Environmental noise levels in the rooms were perceptually judged to be low and acceptable for audio recording and analysis. Spontaneous cries of each infant less than 30 min before feeding were recorded for more than 60 s using a wave recorder at a 44.1 kHz sampling rate with 16-bit quantization. If an infant cried for longer than 60 s, the 60 s period of successive cries nearest to the feeding were selected. During recording, the distance between the microphone and the infant’s mouth was approximately 15 cm (EDIROL R-09; Roland, Corp., Los Angeles, CA, United States).

#### Assessment of Language Development at a Corrected Age of 18 months

The children were enrolled in a follow-up session to assess language development at 18 months of corrected age. Language development was evaluated using the MacArthur Communicative Development Inventory, adapted for Japanese (MCDI; Words and Gestures; Ogura and Watamaki, 1998) and the Kyoto Scale of Psychological Development (KSPD; Ikuzawa et al., 2002). The MCDI is a valid, reliable parent survey for assessing early communication and language development from 8 to 18 months. We used the questionnaire to evaluate infants’ gesture, receptive, and expressive language skills. Primary caregivers completed the MCDI after the administration of the KSPD. The KSPD is a Japanese standardized developmental scale commonly administered to typically developing infants and low-functioning children with disabilities. This scale is an individualized face-to-face test based on Gesell’s developmental diagnosis and the assessment items of the Binet test. The KSPD measures general developmental progress and delays in three domains: postural-motor (P-M), cognitive-adaptive (C-A), and language-social (L-S). The developmental quotients of these three areas are highly correlated with corresponding composite scores (i.e., motor, cognitive, and language) on the Bayley III (Kono et al., 2016). The KSPD was conducted in an examination room at Kyoto University Hospital for preterm infants and in an experimental room at Kyoto University for term infants. Visual distractions were removed when possible and the environmental noise level of the room was perceptually judged to be low.

For the follow-up assessment at 18 months, 83 infants from the initial cohort (*n* = 107) completed the MCDI (VP group: *n* = 29, MLP group: *n* = 27, and term group: *n* = 19) and/or KSPD (VP group: *n* = 30, MLP group: *n* = 32, and term group: *n* = 20). At the MCDI assessment, the mean corrected age of the VP group was 18.63 months (*SD* = 0.50, range = 17.71–19.81), that of the MLP group was 18.43 months (*SD* = 0.57, range = 17.45–19.91), and that of the term group was 18.45 months (*SD* = 0.44, range = 17.58–19.12). At the KSPD assessment, the mean corrected age of the VP group was 18.47 months (*SD* = 0.31, range = 17.71–19.19), that of the MLP group was 18.33 months (*SD* = 0.55, range = 17.45–19.68), and that of the term group was 18.17 months (*SD* = 0.42, range = 17.15–18.89). A number of participants did not complete the follow-up assessments for the following reasons: refusal to participate (e.g., moving far away; *n* = 9), incomplete questionnaire and developmental tests (*n* = 3), and no need to visit the hospital due to a lack of clinical conditions for the preterm group (*n* = 12). The included group (*n* = 83, female: *n* = 45) and the excluded group (*n* = 24, female: *n* = 14) did not significantly differ in terms of the acoustic features of the cries and the developmental outcomes. However, compared to the excluded group, the included group had a significantly earlier gestational age (included group: *M* = 33.5 weeks, *SD =* 4.8, range = 23.1–41.4; excluded group: *M* = 35.5 weeks, *SD* = 4.1, range = 26.6–40.7; *t*_105_ = -1.89, *d* = 0.44, *p* = 0.046). These group differences may have been due to the fact that the excluded group had a relatively high ratio of term infants and MLP preterm infants with fewer clinical problems.

### Data Analysis

#### Cry Acoustic Features

Acoustic features of infants’ cries were assessed using Praat (ver. 6.0.19) (Boersma and Weenink, 2016). A cry utterance was defined as a vocal output occurring on a single expiration and lasting for at least 0.3 s, to exclude non-cry sounds, such as coughs (Shinya et al., 2014, 2016). We manually segmented each infant’s 60 s cry series into single cry utterances based on amplitude-by-time waveforms, and 4,012 cries were extracted in total. These onset and offset points were also estimated to determine the duration of each cry utterance. Based on visual inspections of spectrograms, cry utterances containing broad regions of environmental noise were excluded from analysis to avoid artifacts when determining the *F_0_*. Additionally, phonatory noise phenomena and phenomena such as sudden *F_0_* shifts or subharmonics were excluded from the analyses because the *F_0_* contour cannot be reliably determined in those signals. Ultimately, 3,578 cries (89.2% of all cry utterances) were used in the acoustic analyses (number of cries per infant; VP group: *M* = 38.6 [*SD* = 16.7]; MLP group: *M* = 39.0 [*SD* = 17.3]; and term group: *M* = 34.1 [*SD* = 13.9]); a one-way analysis of variance (ANOVA) revealed no significant differences among the three groups (*p* = 0.401).

The cry utterances were down-sampled to 22.05 kHz and low-pass filtered at 10 kHz to eliminate outliers and artifacts. The following *F_0_* estimation was calculated for each cry utterance using a Praat autocorrelation algorithm (a noise-resistant autocorrelation method at 150–900 Hz with a Hanning window length of 0.05 s) and then averaged for each infant. The *F_0_* min (lowest *F_0_* of a cry utterance), *F_0_* mean (arithmetic average of *F_0_*), and *F_0_* max (highest *F_0_*) were calculated to assess the height of *F_0_*. These *F_0_* measures were previously reported by our research group (Shinya et al., 2014, 2016); the values and group differences of the *F_0_* measures and cry duration are provided in Supplementary Table S1.

The height of the *F_0_* variation was estimated to assess variability in the cry melody. As a measure of *F_0_* variation within cry utterance, *F_0_* range (difference between *F_0_* min and *F_0_* max) and *F_0_* sigma (*SD* for the mean *F_0_*) were calculated. Furthermore, the *F_0_* transposition range was determined to assess *F_0_* variation within 60 s cry series. Beginning in the neonatal period, infants produce shape-similar cry melodies at different frequency levels for which there are large individual differences in the transposition range, even though the range gradually increases with postnatal age (Wermke and Mende, 2009). Based on these observations, *F_0_* transposition range was calculated by subtracting the minimum value of *F_0_* max from the maximum value of *F_0_* max within cry series (**Figure 1C**).

To assess the complexity of cry melody, the melody complexity index (MCI) was calculated (Wermke et al., 2007, 2014). Neonatal cries with an identifiable melody commonly exhibit a single ascending–descending arc. These simple single-arc melodies are increasingly replaced by complex melodies (i.e., double- or multiple-arc melodies) over the first several months in term infants (Wermke et al., 2002, 2007). Thus, cry utterances were subdivided into those with only a simple (single-arc) melody and those with a complex (multiple-arc) melody (**Figures 1A,B**). Furthermore, arc-like melody substructures were identified, with arcs defined as longer than 150 ms and exhibiting an *F_0_* range of at least two semitones (Wermke et al., 2014). MCI ratio was calculated for each infant by dividing the number of cries consisting of multiple-arc melodies by the total number of cry utterances (Wermke et al., 2007, 2014). In addition, we calculated an MCI number for each infant by averaging the number of arcs within cry utterance. Because some infants exhibit no-arc melodies (i.e., “flat plateau”; Wermke and Mende, 2009) or more than double-arc (**Figures 1A,B**) melodies during the neonatal period, the MCI number is assumed to be more sensitive for assessing the ability to produce complex cry melodies compared to the MCI ratio.

#### Language Development

For the MCDI, the following three sub-components of early communication and language development were calculated at 18 months of corrected age: gesture, receptive language, and expressive language. Because the expressive language score was skewed positively, it was transformed using a natural log (*ln*) transform to normalize the distribution for the statistical analysis. For the KSPD, the developmental quotient was calculated by dividing developmental age by corrected age and then multiplying the resulting quotient by 100 for each of three areas (i.e., P-M, C-A, and L-S). It was expected that the melodic features of spontaneous cries would be selectively associated with the L-S score, which is a measure of early communication and language development on the KSPD.

## Results

### Differences of Cry Melodic Features and Developmental Outcomes between Preterm and Term Infants

**FIGURE 2:**
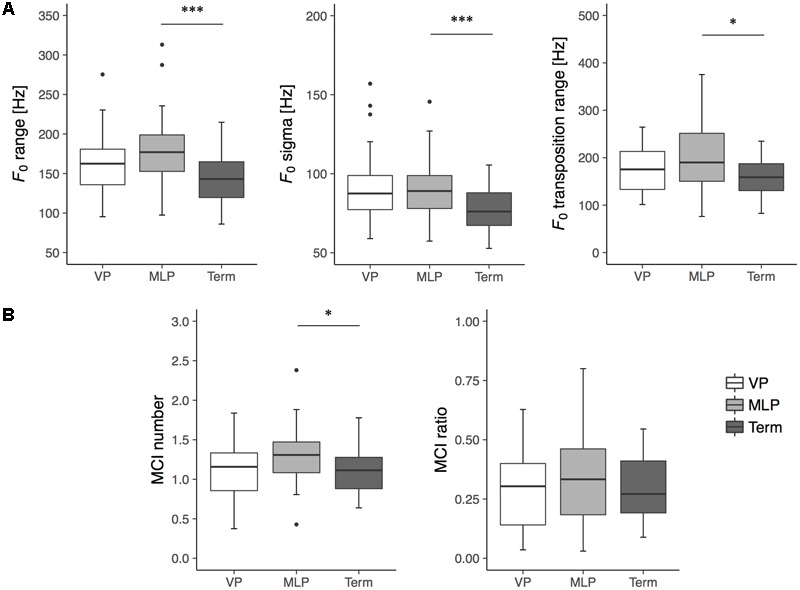
Box plots for melodic features of spontaneous crying: **(A)** variability indexes (i.e., *F_0_* range, *F_0_* sigma, *F_0_* Transposition range) and **(B)** melody complexity indexes (i.e., MCI ratio, MCI number) in very preterm (VP; *n* = 36, white box), moderate-to-late preterm (MLP; *n* = 41, gray box), and term infants (Term; *n* = 30, black box) at term-equivalent age. Boxes represent medians with upper and lower quartiles; whiskers represent maximum and minimum values, excluding outliers indicated by circles, at least 1.5 times the interquartile range (i.e., 1.5 box lengths from the upper or lower edge of the box). ^∗^*p* < 0.05; ^∗∗∗^*p* < 0.01.

The acoustic features of spontaneous crying at term-equivalent age are detailed in **Table 1** and language developmental scores at 18 months of corrected age are provided in **Table 2**. For the melodic features of crying, a one-way ANOVA assessing data from the gestational group revealed significant group differences in *F_0_* range, *F_0_* sigma, *F_0_* transposition range, and MCI number (**Table 1**). *Post hoc* testing using the Bonferroni method revealed that the MLP group had higher values for *F_0_* range (*p* < 0.001), *F_0_* sigma (*p* < 0.001), *F_0_* transposition range (*p* = 0.011), and MCI number (*p* = 0.041) than the term group (**Figure 2**).

**Table 2:** Developmental outcomes in preterm and term infants at 18 months old of corrected age.

	Preterm infants						Term infants					
	VP			MLP			Term					
	*M*	*SD*	*Range*	*M*	*SD*	*Range*	*M*	*SD*	*Range*	*F*-Value	*p*-Value	*Post hoc* (*p* < 0.05)
**MCDI**	**(*n =* 29)**			**(*n =* 27)**			**(*n =* 19)**					
Gesture	35	11	9–51	40	9	21–53	41	6	26–51	4.06	0.021	VP < Term
Receptive language	127	81	10–297	236	95	43–410	153	81	46–300	11.93	<0.001	VP < MLP, MLP > Term
Expressive language	24	30	0–134	63	61	1–218	38	40	1–170	4.99	0.009	VP < MLP
**KSPD**	**(*n =* 30)**			**(*n =* 32)**			**(*n =* 20)**					
Postural-motor	96	22	28–151	105	20	55–156	101	15	64–129	1.69	0.191	
Cognitive-adaptation	96	15	60–121	104	10	81–127	108	10	81–123	7.75	<0.001	VP < MLP, VP < Term
Language-social	92	14	62–116	102	15	54–125	103	11	75–120	5.62	0.005	VP < MLP, VP < Term


*VP, very preterm; MLP, moderate to late preterm; MCDI, MacArthur Communicative Development Inventory; KSPD, Kyoto Scale of Psychological Development.*

For the developmental scores, there were significant group differences in the MCDI scores for gesture, receptive language, and expressive language and KSPD scores for the C-A and L-S (**Table 2**). *Post hoc* testing of the MCDI data showed that the VP group had lower gesture scores than the term group (*p* = 0.046) as well as lower receptive and expressive language scores than the MLP group (*p* < 0.001). Additionally, the receptive language score of the MLP group was higher than that of the term group (*p* = 0.023). For the KSPD, the VP group exhibited a lower C-A score than the MLP and term groups (*p* = 0.035; *p* = 0.003, respectively) and the L-S score of the VP group was lower than that of the MLP group (*p* = 0.041).

### Relations between Cry Melodic Features and Language Development

**FIGURE 3:**
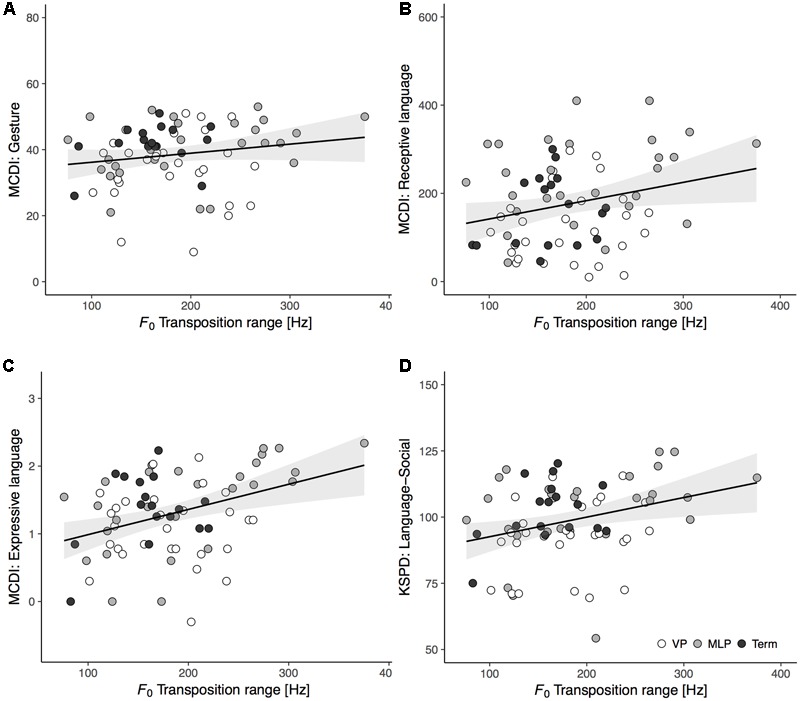
Scatter plots and regression lines with 95% confidence intervals showing the relationships between *F_0_* transposition range of spontaneous cries at term-equivalent age and language outcomes at 18 months of corrected age: **(A)** MCDI’s gesture (*n* = 75, *r* = 0.18, *p* = 0.119); **(B)** MCDI’s receptive language (*n* = 75, *r* = 0.25, *p* = 0.033); **(C)** MCDI’s expressive language (*n* = 75, *r* = 0.38, *p* < 0.001); **(D)** KSPD’s Language-Social (*n* = 82, *r* = 0.29, *p* = 0.008). The groups of infants were VP (white circles), MLP (gray circles), and Term (black circles).

We firstly performed Pearson’s correlation to assess relations between cry melodic features at term and developmental outcomes at 18 months for all participants (MCDI: *n* = 75; KSPD: *n* = 82). The *F_0_* transposition range exhibited a significant positive correlation with the receptive language (*r* = 0.25, *p* = 0.033) and expressive language (*r* = 0.38, *p* < 0.001) scores on the MCDI and with the L-S score (*r* = 0.29, *p* = 0.008) on the KSPD; there were no significant correlations with the MCDI gesture score (*r* = 0.18, *p* = 0.119) or the KSPD P-M and C-A scores (*r* = 0.04, *p* = 0.729; *r* = 0.18, *p* = 0.114, respectively). Scatter plots were constructed to illustrate the correlations between *F_0_* transposition range at term and language development at 18 months (**Figure 3**). The other acoustic variables did not significantly relate to any developmental outcomes (Supplementary Table S2).

Additionally, hierarchical multiple regression analyses were conducted to analyze further the independent contributions of cry melodic variables to developmental outcomes, especially language development. Gestational age (Aarnoudse-Moens et al., 2009) and IUGR (Bergvall et al., 2006) were entered as independent variables in the first two steps; the next two steps included weight at cry recording and corrected age of developmental assessment, and the final step included each of the melodic variables of crying. In this model, we excluded other demographic variables related to gestational age (i.e., birth weight, Apgar score at 5 min, and postnatal age at cry recording) to avoid any collinearity of the predictors.

In the first four steps, a shorter gestational age significantly predicted lower gesture scores on the MCDI (*p* < 0.001) and lower P-M (*p* = 0.033), C-A (*p* < 0.001), and L-S (*p* = 0.004) scores on the KSPD. IUGR was significantly related to lower gesture scores on the MCDI (*p* = 0.018) and C-A (*p* = 0.005) and L-S (*p* = 0.018) scores on the KSPD. The next two steps did not result in significant values for any developmental outcomes (**Table 3**). In the final step, a higher *F_0_* transposition range predicted higher gesture (*p* = 0.022), receptive language (*p* = 0.008), and expressive language (*p* < 0.001) scores on the MCDI and C-A (*p* = 0.024) and L-S (*p* < 0.001) scores on the KSPD (**Table 3**). The other melodic features of crying and *F_0_* measures did not significantly predict any developmental outcomes; the partial correlation coefficients are reported in Supplementary Table S2.

**Table 3:** Hierarchical multiple regression analyses predicting developmental outcomes in all participants.

		MacArthur Communicative Development Inventory (*n* = 75)								
		Gesture			Receptive language			Expressive language		
Step	Predictor	*β*	*ΔR*^*2*^	*ΔF*	*β*	*ΔR*^*2*^	*ΔF*	*β*	*ΔR*^*2*^	*ΔF*
1	Gestational age	0.35	0.12	10.31***	0.20	0.04	3.1	0.22	0.05	3.85
2	IUGR	-0.26	0.07	5.91*	-0.08	0.01	0.47	-0.20	0.04	3.16
3	Weight at cry recording	-0.16	0.02	1.79	-0.23	0.04	3.25	-0.03	0.00	0.06
4	Corrected age at developmental assessment	-0.14	0.02	1.82	-0.13	0.02	1.26	0.02	0.00	0.03
5	*F_0_* transposition range	0.24	0.06	5.53*	0.30	0.09	7.49**	0.40	0.16	14.99***
	*R^2^* total =	0.29, *F*_5,69_ = 5.56, *p* < 0.001			19, *F*_5,69_ = 3.30, *p* = 0.010			0.25, *F*_5,69_ = 4.68, *p* < 0.001		

		**Kyoto Scale of Psychological Development (*n* = 82)**								
		**Postural-motor**			**Cognitive-adaptation**			**Language-social**		
**Step**	**Predictor**	***β***	***ΔR*^*2*^**	***ΔF***	***β***	***ΔR*^*2*^**	***ΔF***	***β***	***ΔR*^*2*^**	***ΔF***

1	Gestational age	0.24	0.06	4.69*	0.49	0.24	25.07***	0.32	0.10	8.95**
2	IUGR	-0.00	0.00	0.00	-0.27	0.07	8.29**	-0.25	0.06	5.87*
3	Weight at cry recording	0.04	0.00	0.09	0.09	0.01	0.66	-0.15	0.02	1.64
4	Corrected age at developmental assessment	-0.16	0.02	1.95	-0.02	0.00	0.05	-0.14	0.02	1.63
5	*F_0_* transposition range	0.08	0.01	0.53	0.22	0.04	5.31*	0.36	0.12	13.55***
	*R^2^* total =	0.09, *F_576_ =* 1.51, *p* = 0.197			0.36, *F_576_ =* 8.62, *p <* 0.001			0.32, *F_576_ =* 7.10, *p <* 0.001		

*β, standardized regression coefficient; IUGR, intrauterine growth retardation ^∗^*p* < 0.05, ^∗∗^*p* < 0.01, ^∗∗∗^*p* < 0.001.*

## Discussion

To the best of our knowledge, the present study is the first to investigate the effects of preterm birth on melodic features (i.e., time variation of *F_0_*) of spontaneous cries at term-equivalent age. Additionally, the relationships of the analyzed melodic features of crying with language outcomes at 18 months of corrected age were examined in a sample including preterm and term infants, based on the assumption of cry melody as an ontogenetic origin of language (Darwin, 1872; Lieberman, 1985; Wermke and Mende, 2009). The present results revealed that the spontaneous cries of the MLP group had significantly higher *F_0_* variation and melody complexity compared to term infants, and that increased *F_0_* variation within cry series (i.e., *F_0_* transposition range) at term was significantly associated with better language and cognitive outcomes at 18 months. Even after confounding factors such as gestational age and IUGR were controlled for, the *F_0_* transposition range of spontaneous crying significantly predicted language and cognitive outcomes, particularly expressive language skill.

We demonstrated that the spontaneous cries of MLP infants at term showed higher *F_0_* variation (i.e., *F_0_* range, *F_0_* sigma, and *F_0_* transposition range) and melody complexity (i.e., MCI number) than those of term infants. In contrast, there were no significant differences in melodic features between VP and term infants. Previous studies of term infants have reported that the melody of spontaneous cries gradually exhibits increased variability (Prescott, 1975) and complexity (Wermke et al., 2002, 2007) across the first several months. These developmental changes are assumed to reflect the maturation of vocal control over melodic contours in spontaneous crying (Wermke and Mende, 2009). Additionally, because preterm infants begin vocalizing as early as 8 weeks before their projected due date (Caskey et al., 2011), the postnatal experiences of spontaneous crying at term in preterm infants may be greater than that of term infants. Therefore, the additional vocal experience of the MLP group in the extrauterine environment may facilitate vocal control over melodic contours during spontaneous crying. Actually, this observation may be in line with previous research reporting that healthy preterm infants, with age corrected for gestational age, began canonical babbling earlier than term infants (Eilers et al., 1993). On the other hand, it is possible that this additional experience might not contribute to the increased variability and complexity of cry melodies in the VP group due to their more complicated neurophysiological states (Peterson et al., 2000; Aarnoudse-Moens et al., 2009; Naoi et al., 2013; Shinya et al., 2016).

Moreover, the present study showed that a larger *F_0_* variation within cry series (i.e., *F_0_* transposition range) of spontaneous crying at term was significantly related to better language skills at 18 months. This finding is consistent with the notion of a potential continuity between the development of cry melody and language acquisition (Darwin, 1872; Lieberman, 1985; Wermke and Mende, 2009). Notably, the larger range was primarily associated with higher levels of expressive language skills at 18 months among developmental outcomes. *F_0_* transposition range in infant crying reflects the ability to transpose melody across different frequency levels. Although the range is reported to increase gradually with postnatal age, there are large individual differences in *F_0_* transposition range even during the neonatal period (Wermke and Mende, 2009). Thus, individual differences in *F_0_* transposition range may reflect voluntary control over cry melody, which, in turn, contributes to later non-cry vocalizations such as babbling and expressive language (Lieberman, 1985). In practice, during language acquisition, the ability to transpose a melody across different frequency levels is assumed to be necessary for imitating simple melodies and speech sounds in one’s ambient language beginning in the neonatal period (Kuhl and Meltzoff, 1996; Mampe et al., 2009; Wermke and Mende, 2009; Gratier and Devouche, 2011; Wermke et al., 2016). Importantly, the effects of *F_0_* transposition range at term on language development at 18 months are independent of confounding factors such as gestational age and IUGR (Hille et al., 2007; Aarnoudse-Moens et al., 2009). Considering that preterm infants, especially VP infants, have higher risks of later language-related problems (Foster-Cohen et al., 2007; Luu et al., 2009; Barre et al., 2011; Sansavini et al., 2011; van Noort-van der Spek et al., 2012), the *F_0_* transposition range of spontaneous cries will be an additional measure that can predict the risks in preterm peers. Nevertheless, further studies are needed to replicate this preliminary observation of a relationship between the *F_0_* transposition range of spontaneous cries and later language acquisition.

The present study did not reveal any significant associations between melody complexity at term and language outcomes at 18 months, which is inconsistent with previous reports showing that MCI rate during the second month is positively related to language outcomes at 2.5 years in term children (Wermke et al., 2007). The discrepancy might be due to the difference in the definitions of MCI. Wermke et al. (2007) used for a single melody arc definition an amplitude of at least three semitones, while the present study used only two semitones (Wermke et al., 2014). This different approach may increase the number of identifiable arcs and affect the results of the associations between MCI and language outcomes at 18 months. On the other hand, melody in spontaneous cries becomes increasingly more complex during the first several months of life (Wermke et al., 2002, 2007). Therefore, the melody complexity of spontaneous cries is assumed to be much lower at term age than at the second month. In fact, the mean MCI ratio at term was 0.29 in the present study while Wermke et al. (2007) found this ratio to be 0.52 during the second month. Thus, it is possible that melody complexity at term age has not sufficiently developed to reflect individual differences in voluntary vocal control that later contribute to language acquisition at 18 months of age. Future studies should follow-up the development of melody complexity of spontaneous cries in preterm peers after the term period and relate these findings to language development.

Another important finding is that the *F_0_* transposition range of spontaneous crying at term was positively associated with cognitive development at 18 months, as indexed by the C-A score on the KSPD. One possibility is that individual differences in *F_0_* transposition range at term may reflect the integrity of the developing CNS in a manner similar to that of spontaneous physical movements, such as general movements (GMs; Prechtl, 2001). Particularly in preterm infants, abnormal GMs at an early developmental stage (i.e., less variability and complexity) are a major predictor of cerebral palsy (Prechtl, 2001) and has been recently reported to relate to delayed cognitive development from infancy to school age (Butcher et al., 2009; Beccaria et al., 2012; Kanemaru et al., 2013). Less variable and frequent spontaneous movements are assumed to be due to reductions in the modulation of the generation of spontaneous movements from central pattern generators in the brainstem by the developing cerebral cortex; this can result in domain general developmental delays (Einspieler et al., 2016). Spontaneous crying is also generated from the reflexive central pattern generators in the periaqueductal gray and nucleus retroambiguus. These brainstem regions may be modulated by forebrain regions such as the amygdala and anterior cingulate cortex and lead to more variable patterns of melody in infant crying (Newman, 2007; Holstege and Subramanian, 2015). Thus, the limited *F_0_* transposition range in spontaneous crying at term might reflect the reduced integrity of the developing CNS, which may be a partial risk factor of cognitive delay (Butcher et al., 2009; Einspieler et al., 2016).

The present study has several limitations that should be noted. First, the selection of participants for the present study might reflect a sample bias because the sample size was relatively small, especially for the term group. Future research is needed to replicate these findings using a larger sample that controls for confounding factors, such as socioeconomic status (Eilers et al., 1993; Fernald et al., 2012) and exposure to another language (Hoff et al., 2011), which may potentially affect developmental outcomes, including language development. Second, the present study did not investigate the melodic features of spontaneous cries in preterm infants from birth onward through development. To examine further the possibility that additional vocal experience may facilitate the development of cry melodies in healthy preterm peers, a longitudinal assessment of the developmental process from birth to term age will be required. Third, language development was evaluated only at 18 months of corrected age. In the present study, the difference in language development between the preterm and term groups at that timepoint was smaller than that observed in previous studies (Foster-Cohen et al., 2007; Sansavini et al., 2011). This may be because the present study included only preterm peers without severe complications and because the relationships between low birth weight and cognitive outcomes have been mitigated by recent medical advances (Goisis et al., 2017). However, researchers also need to follow-up with language outcomes after 18 months, considering that several recent studies have reported preterm children exhibit higher risks of language-related problems after infancy (Luu et al., 2009; Barre et al., 2011; van Noort-van der Spek et al., 2012).

## Conclusion

The present study revealed that MLP infants exhibited greater *F_0_* variation and complexity in neonatal spontaneous crying at term age compared to term infants, although the differences were not found between VP and term infants. Furthermore, *F_0_* variation within cry series (i.e., *F_0_* transposition range) was positively related to language development, particularly expressive language skills, at 18 months, which is consistent with previous observations of the potential link between cry melody and language (Darwin, 1872; Lieberman, 1985; Wermke and Mende, 2009). The present findings suggest that the additional postnatal vocal experience of MLP infants increased *F_0_* variation and complexity of spontaneous cries at term. Additionally, the increased *F_0_* variation within cry series may partially reflect developing voluntary vocal control, which contributes to expressive language in infancy. Future studies should examine whether assessments of the melodic features of crying represent a valuable addition to existing early indices and improve the ability to predict language outcomes in preterm peers.

## Author Contributions

YS, MK, FN, MI, and MM contributed to the conception and design of the work; contributed in revising the work for important intellectual content. YS, FN, and MI contributed in the data acquisition. YS, MK, and MM contributed in the analysis and interpretation of data for the work. YS and MI contributed in drafting the work. All authors approved the final version to be published, and agree to be accountable for all aspects of the work in ensuring that questions related to the accuracy or integrity of any part of the work are appropriately investigated and resolved.

## Conflict of Interest Statement

The authors declare that the research was conducted in the absence of any commercial or financial relationships that could be construed as a potential conflict of interest.

## References

[B1] Aarnoudse-Moens C. S. H. Weisglas-Kuperus N. van Goudoever J. B. Oosterlaan J. (2009). Meta-analysis of neurobehavioral outcomes in very preterm and/or very low birth weight children. *Pediatrics* 124 717–728. 10.1542/peds.2008-2816 19651588

[B2] Barre N. Morgan A. Doyle L. W. Anderson P. J. (2011). Language abilities in children who were very preterm and/or very low birth weight: a meta-analysis. *J. Pediatr.* 158 766–774. 10.1016/j.jpeds.2010.10.032 21146182

[B3] Beccaria E. Martino M. Briatore E. Podestà B. Pomero G. Micciolo R. (2012). Poor repertoire general movements predict some aspects of development outcome at 2years in very preterm infants. *Early Hum. Dev.* 88 393–396. 10.1016/j.earlhumdev.2011.10.002 22044887

[B4] Bergvall N. Iliadou A. Johansson S. Tuvemo T. Cnattingius S. (2006). Risks for low intellectual performance related to being born small for gestational age are modified by gestational age. *Pediatrics* 117 e460–e467. 10.1542/peds.2005-0737 16510624

[B5] Boersma P. Weenink D. (2016). *Praat: do0069ng Phonetics by Computer (v. 6.0.19). [Computer Program.].* Available at: http://www.praat.org

[B6] Butcher P. R. van Braeckel K. Bouma A. Einspieler C. Stremmelaar E. F. Bos A. F. (2009). The quality of preterm infants’ spontaneous movements: an early indicator of intelligence and behaviour at school age. *J. Child Psychol. Psychiatry* 50 920–930. 10.1111/j.1469-7610.2009.02066.x 19457048

[B7] Cacace A. T. Robb M. P. Saxman J. H. Risemberg H. Koltai P. (1995). Acoustic features of normal-hearing pre-term infant cry. *Int. J. Pediatr. Otorhinolaryngol.* 33 213–224. 10.1016/0165-5876(95)01211-7 8557478

[B8] Caskey M. Stephens B. Tucker R. Vohr B. (2011). Importance of parent talk on the development of preterm infant vocalizations. *Pediatrics* 128 910–916. 10.1542/peds.2011-0609 22007020

[B9] Darwin C. (1871). *The Descent of Man, and Selection in Relation to Sex.* London: John Murray.

[B10] Darwin C. (1872). *The Expression of Emotion in Man and Animals.* London: John Murray.

[B11] De Schuymer L. De Groote I. Beyers W. Striano T. Roeyers H. (2011a). Preverbal skills as mediators for language outcome in preterm and full term children. *Early Hum. Dev.* 87 265–272. 10.1016/j.earlhumdev.2011.01.029 21330069

[B12] De Schuymer L. De Groote I. Striano T. Stahl D. Roeyers H. (2011b). Dyadic and triadic skills in preterm and full term infants: a longitudinal study in the first year. *Infant Behav. Dev.* 34 179–188. 10.1016/j.infbeh.2010.12.007 21185604

[B13] Eilers R. E. Oller D. K. Levine S. Basinger D. Lynch M. P. Urbano R. (1993). The role of prematurity and socioeconomic status in the onset of canonical babbling in infants. *Infant Behav. Dev.* 16 297–315. 10.1016/0163-6383(93)80037-9

[B14] Einspieler C. Bos A. F. Libertus M. E. Marschik P. B. (2016). The general movement assessment helps us to identify preterm infants at risk for cognitive dysfunction. *Front. Psychol.* 7:406. 10.3389/fpsyg.2016.00406 27047429 PMC4801883

[B15] Fernald A. Marchman V. A. Weisleder A. (2012). SES differences in language processing skill and vocabulary are evident at 18 months. *Dev. Sci.* 16 234–248. 10.1111/desc.12019 23432833 PMC3582035

[B16] Foster-Cohen S. Edgin J. O. Champion P. R. Woodward L. J. (2007). Early delayed language development in very preterm infants: evidence from the MacArthur-Bates CDI. *J. Child Lang.* 34 655–675. 10.1017/S0305000907008070 17822143

[B17] Garner P. W. Landry S. H. Richardson M. A. (1991). The development of joint attention skills in very-low-birth-weight infants across the first 2 years. *Infant Behav. Dev.* 14 489–495. 10.1016/0163-6383(91)90035-Q

[B18] Goisis A. Özcan B. Myrskylä M. (2017). Decline in the negative association between low birth weight and cognitive ability. *Proc. Natl. Acad. Sci. U.S.A.* 114 84–88. 10.1073/pnas.1605544114 27994141 PMC5224382

[B19] Gratier M. Devouche E. (2011). Imitation and repetition of prosodic contour in vocal interaction at 3 months. *Dev. Psychol.* 47 67–76. 10.1037/a0020722 21244150

[B20] Hoff E. Core C. Place S. Rumiche R. Senor M. Parra M. (2011). Dual language exposure and early bilingual development. *J. Child Lang.* 39 1–27. 10.1017/S0305000910000759 21418730 PMC4323282

[B58] Hille E. T. M. Weisglas-Kuperus N. van Goudoever J. B. Jacobusse G. W. Ens-Dokkum M. H. de Groot L. (2007). Functional outcomes and participation in young adulthood for very preterm and very low birth weight infants: the Dutch project on preterm and small for gestational age infants at 19 years of age. *Pediatrics* 120: e587–95. 17766499 10.1542/peds.2006-2407

[B21] Holstege G. Subramanian H. H. (2015). Two different motor systems are needed to generate human speech. *J. Comp. Neurol.* 524 1558–1577. 10.1002/cne.23898 26355872

[B22] Ikuzawa M. Matsushita H. Nakase A. (2002). *Kyoto Scale Psychological Development 2001.* Kyoto: Kyoto International Social Welfare Exchange Centre.

[B23] Imafuku M. Kawai M. Niwa F. Shinya Y. Inagawa M. Myowa-Yamakoshi M. (2017). Preference for dynamic human images and gaze-following abilities in preterm infants at 6 and 12 months of age: an eye-tracking study. *Infancy* 22 223–239. 10.1111/infa.12144 33158339

[B24] Jespersen O. (1922). *Language: Its Nature, Development, and Origin.* London: George Allen and Unwin Ltd.

[B25] Johnston C. C. Stevens B. Craig K. D. Grunau R. V. (1993). Developmental changes in pain expression in premature, full-term, two-and four-month-old infants. *Pain* 52 201–208. 10.1016/0304-3959(93)90132-9 8455968

[B26] Jürgens U. (2002). Neural pathways underlying vocal control. *Neurosci. Biobehav. Rev.* 26 235–258. 10.1016/S0149-7634(01)00068-9 11856561

[B27] Kanemaru N. Watanabe H. Kihara H. Nakano H. Takaya R. Nakamura T. (2013). Specific characteristics of spontaneous movements in preterm infants at term age are associated with developmental delays at age 3 years. *Dev. Med. Child Neurol.* 55 713–721. 10.1111/dmcn.12156 23601036

[B28] Kisilevsky B. S. Hains S. M. J. Brown C. A. Lee C. T. Cowperthwaite B. Stutzman S. S. (2009). Fetal sensitivity to properties of maternal speech and language. *Infant Behav. Dev.* 32 59–71. 10.1016/j.infbeh.2008.10.002 19058856

[B29] Kono Y. Yonemoto N. Kusuda S. Hirano S. Iwata O. Tanaka K. (2016). Developmental assessment of VLBW infants at 18 months of age: a comparison study between KSPD and Bayley III. *Brain Dev.* 38 377–385. 10.1016/j.braindev.2015.10.010 26542468

[B30] Kuhl P. K. Meltzoff A. N. (1996). Infant vocalizations in response to speech: vocal imitation and developmental change. *J. Acoust. Soc. Am.* 100 2425–2438. 10.1121/1.417951 8865648 PMC3651031

[B31] Kujala A. Huotilainen M. Hotakainen M. Lennes M. Parkkonen L. Fellman V. (2004). Speech-sound discrimination in neonates as measured with MEG. *Neuroreport* 15 2089–2092. 10.1097/00001756-200409150-00018 15486487

[B32] Lieberman P. (1985). “The physiology of cry and speech in relation to linguistic behavior,” in *Infant Crying: Theoretical and Research Perspectives* eds Lester B. M. Boukydis C. F. Z. (New York, NY: Plenum Press) 29–57.

[B33] Luu T. M. Vohr B. R. Schneider K. C. Katz K. H. Tucker R. Allan W. C. (2009). Trajectories of receptive language development from 3 to 12 years of age for very preterm children. *Pediatrics* 124 333–341. 10.1542/peds.2008-2587 19564317 PMC2704989

[B34] Mampe B. Friederici A. D. Christophe A. Wermke K. (2009). Newborns’ cry melody is shaped by their native language. *Curr. Biol.* 19 1994–1997. 10.1016/j.cub.2009.09.064 19896378

[B35] Michelsson K. Järvenpää A. L. Rinne A. (1983). Sound spectrographic analysis of pain cry in preterm infants. *Early Hum. Dev.* 8 141–149. 10.1016/0378-3782(83)90070-1 6884256

[B36] Müller-Preuss P. Jürgens U. (1976). Projections from the “cingular” vocalization area in the squirrel monkey. *Brain Res.* 103 29–43. 10.1016/0006-8993(76)90684-3 56207

[B37] Naoi N. Fuchino Y. Shibata M. Niwa F. Kawai M. Konishi Y. (2013). Decreased right temporal activation and increased interhemispheric connectivity in response to speech in preterm infants at term-equivalent age. *Front. Psychol.* 4:94. 10.3389/fpsyg.2013.00094 23459601 PMC3585712

[B38] Newman J. D. (2007). Neural circuits underlying crying and cry responding in mammals. *Behav. Brain Res.* 182 155–165. 10.1016/j.bbr.2007.02.011 17363076 PMC1995563

[B39] Ogura T. Watamaki T. (1998). *Research and Development of Early Language Development Inventory.* Kobe: Kobe University.

[B40] Partanen E. Kujala T. Näätänen R. Liitola A. Sambeth A. Huotilainen M. (2013). Learning-induced neural plasticity of speech processing before birth. *Proc. Natl. Acad. Sci. U.S.A.* 110 15145–15150. 10.1073/pnas.1302159110 23980148 PMC3773755

[B41] Peña M. Pittaluga E. Mehler J. (2010). Language acquisition in premature and full-term infants. *Proc. Natl. Acad. Sci. USA.* 107 3823–3828. 10.1073/pnas.0914326107 20133589 PMC2840424

[B42] Peterson B. S. Vohr B. Staib L. H. Cannistraci C. J. Dolberg A. Schneider K. C. (2000). Regional brain volume abnormalities and long-term cognitive outcome in preterm infants. *JAMA* 284 1939–1947. 10.1001/jama.284.15.1939 11035890

[B43] Prechtl H. F. (2001). General movement assessment as a method of developmental neurology: new paradigms and their consequences. The 1999 Ronnie MacKeith lecture. *Dev. Med. Child Neurol.* 43 836–842. 10.1017/S0012162201001529 11769272

[B44] Prescott R. (1975). Infant cry sound; developmental features. *J. Acoust. Soc. Am.* 57 1186–1191. 10.1121/1.380577 1127173

[B45] Sansavini A. Guarini A. Savini S. Broccoli S. Justice L. Alessandroni R. (2011). Longitudinal trajectories of gestural and linguistic abilities in very preterm infants in the second year of life. *Neuropsychology* 49 3677–3688. 10.1016/j.neuropsychologia.2011.09.023 21958647

[B46] Shinya Y. Kawai M. Niwa F. Myowa-Yamakoshi M. (2014). Preterm birth is associated with an increased fundamental frequency of spontaneous crying in human infants at term-equivalent age. *Biol. Lett.* 10:20140350. 10.1098/rsbl.2014.0350 25122740 PMC4155907

[B47] Shinya Y. Kawai M. Niwa F. Myowa-Yamakoshi M. (2016). Associations between respiratory arrhythmia and fundamental frequency of spontaneous crying in preterm and term infants at term-equivalent age. *Dev. Psychobiol.* 58 724–733. 10.1002/dev.21412 27037599 PMC5071706

[B48] Smith L. Ulvund S. E. (2003). The role of joint attention in later development among preterm children: linkages between early and middle childhood. *Soc. Dev.* 12 222–234. 10.1111/1467-9507.00230

[B49] Stevens B. McGrath P. Gibbins S. Beyene J. Breau L. Camfield C. (2007). Determining behavioural and physiological responses to pain in infants at risk for neurological impairment. *Pain* 127 94–102. 10.1016/j.pain.2006.08.012 16997468

[B50] van Noort-van der Spek I. L. Franken M. C. Weisglas-Kuperus N. (2012). Language functions in preterm-born children: a systematic review and meta-analysis. *Pediatrics* 129 745–754. 10.1542/peds.2011-1728 22430458

[B51] Wasz-Höckert O. Michelsson K. Lind J. (1985). “Twenty-five years of Scandinavian cry research,” in *Infant Crying: Theoretical and Research Perspectives* eds Lester B. M. Boukydis C. F. Z. (New York, NY: Plenum Press) 83–104.

[B52] Webb A. R. Heller H. T. Benson C. B. Lahav A. (2015). Mother’s voice and heartbeat sounds elicit auditory plasticity in the human brain before full gestation. *Proc. Natl. Acad. Sci. U.S.A.* 112 3152–3157. 10.1073/pnas.1414924112 25713382 PMC4364233

[B53] Wermke K. Hain J. Oehler K. Wermke P. Hesse V. (2014). Sex hormone influence on human infants’ sound characteristics: melody in spontaneous crying. *Biol. Lett.* 10:20140095. 10.1371/journal.pone.0046610 24806423 PMC4046369

[B54] Wermke K. Leising D. Stellzig Eisenhauer A. (2007). Relation of melody complexity in infants’ cries to language outcome in the second year of life: a longitudinal study. *Clin. Linguist. Phon.* 21 961–973. 10.1080/02699200701659243 17972192

[B55] Wermke K. Mende W. (2009). Musical elements in human infants’ cries: in the beginning is the melody. *Music. Sci.* 13 151–175. 10.1177/1029864909013002081 26499296

[B56] Wermke K. Mende W. Manfredi C. Bruscaglioni P. (2002). Developmental aspects of infant’s cry melody and formants. *Med. Eng. Phys.* 24 501–514. 10.1016/S1350-4533(02)00061-9 12237046

[B57] Wermke K. Teiser J. Yovsi E. Kohlenberg P. J. Wermke P. Robb M. (2016). Fundamental frequency variation within neonatal crying: does ambient language matter? *Speech Lang. Hear.* 19 211–217. 10.1080/2050571X.2016.1187903

