# Supplementary material for: Fundamental Frequency Variation of Neonatal Spontaneous Crying Predicts Language Acquisition in Preterm and Term Infants
Source: Front Psychol. 2017 Dec 22;8:2195. doi: 10.3389/fpsyg.2017.02195 (PMC5744644; doi:10.3389/fpsyg.2017.02195)
Supplement: Supplementary file 1 [file Table_S1_and_S2.docx]

Supplementary Material

Fundamental Frequency Variation of Neonatal Spontaneous Crying Predicts Language Acquisition in Preterm and Term Infants

Yuta Shinya^*^, Masahiko Kawai, Fusako Niwa, Masahiro Imafuku, and Masako Myowa-Yamakoshi

*** Correspondence:**Yuta Shinya: [aftertherain428@gmail.com](mailto:shinyayuta428@gmail.com)

This file includes:

- Cry duration and *F_0_* measures of spontaneous cries in preterm and term infants at term-equivalent age (Table S1)
- Pearson's correlations between cry acoustic at term-equivalent age and developmental outcome variables at 18 months of corrected age (Table S2)
